# Supplementary figures and images for: The prevalence of insomnia and restless legs syndrome among Japanese outpatients with rheumatic disease: A cross-sectional study
Source: PLoS One. 2020 Mar 20;15(3):e0230273. doi: 10.1371/journal.pone.0230273 (PMC7083624; doi:10.1371/journal.pone.0230273)

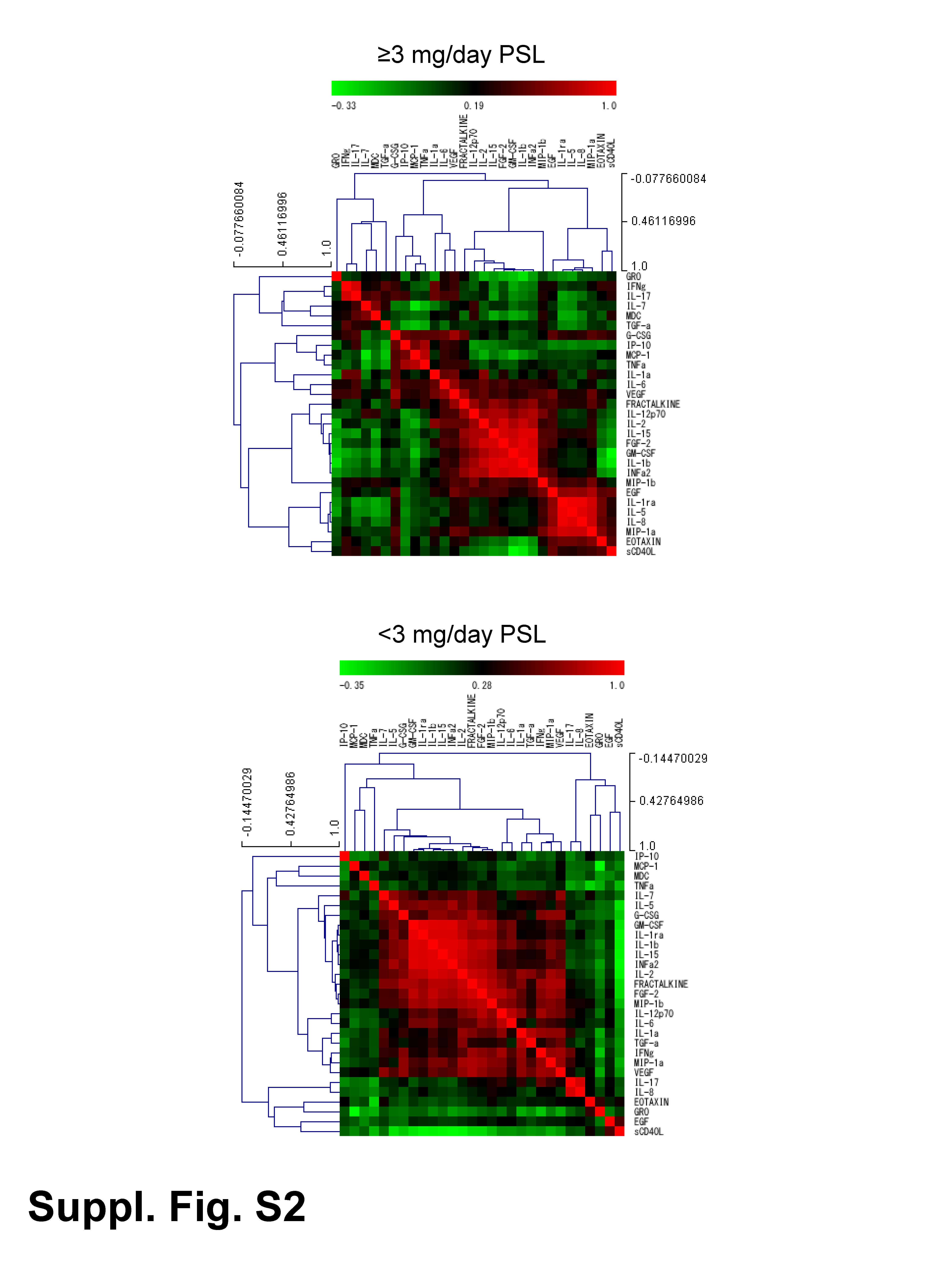

Supplement: S2 Fig — For each cytokine analyzed, the distance between the PSL ≥3 mg/day group and the PSL <3 mg/day group was determined based on Spearman's correlation coefficient. (TIF) [file pone.0230273.s002.tif]
