# Supplementary material for: The prevalence of insomnia and restless legs syndrome among Japanese outpatients with rheumatic disease: A cross-sectional study
Source: PLoS One. 2020 Mar 20;15(3):e0230273. doi: 10.1371/journal.pone.0230273 (PMC7083624; doi:10.1371/journal.pone.0230273)
Supplement: S1 Table — (DOCX) [file pone.0230273.s003.docx]

**Suppl. Table S1.** Insomnia and disease activity scores in the studied RA patients

|  | **Insomnia**  **(n=13)** | **Non-insomnia**  **(n=30)** | **p-value** |
| --- | --- | --- | --- |
| MDGA, median (IQR) | 10.0 (5.0–15.0) | 10.0 (3.0–15.0) | 0.35 |
| TJC28, median (IQR) | 2.0 (1.5–3.5) | 1.0 (0.0–5.0) | 0.41 |
| SJC28, median (IQR) | 3.0 (1.0–4.0) | 0.0 (0.0–2.0) | 0.006 |
| PGA, median (IQR) | 12.0 (5.5–19.0) | 10.0 (4.0–22.0) | 0.50 |
| DAS28-CRP, median (IQR) | 2.6 (2.4–3.4) | 2.1 (1.5–3.2) | 0.12 |
| DAS28-ESR, median (IQR) | 3.4 (2.7–4.5) | 2.5 (1.7–3.9) | 0.09 |
| CDAI, median (IQR) | 7.0 (5.4–13.0) | 3.9 (1.5–11.4) | 0.10 |
| SDAI, median (IQR) | 7.2 (5.8–14.1) | 3.9 (1.6–11.7) | 0.09 |


IQR: interquartile range, MDGA: Medical Doctor's Global Assessment of RA disease activity on a 100-mm visual analog scale, PGA: Patient’s Global Assessment of activity on a 100-mm analog scale, S-insomnia: severe insomnia, SJC28: swollen joint count based on 28-joint assessment, TJC28: tender joint count based on 28-joint assessment.
