# Supplementary material for: The prevalence of insomnia and restless legs syndrome among Japanese outpatients with rheumatic disease: A cross-sectional study
Source: PLoS One. 2020 Mar 20;15(3):e0230273. doi: 10.1371/journal.pone.0230273 (PMC7083624; doi:10.1371/journal.pone.0230273)
Supplement: S2 Table — (DOCX) [file pone.0230273.s004.docx]

**Suppl. Table S2.** Cytokine profile of patients treated with prednisolone (PSL) ≥3 mg/day and those treated with <3 mg/day

|  | | **PSL ≥3 mg/day** | | **PSL <3 mg/day** | **p-value** | | |
| --- | --- | --- | --- | --- | --- | --- | --- |
| IL-1α | 16.2 (0.0–60.7) | | 3.4 (0.0–75.1) | | | 0.99 |  |
| IL-1β | 1.3 (0.0–2.6) | | 1.0 (0.0–6.8) | | | 0.76 |  |
| IL-2 | 0.9 (0.0–6.0) | | 0.7 (0.0–6.4) | | | 0.85 |  |
| IL-5 | 0.2 (0.0–8.6) | | 0.0 (0.0–1.5) | | | 0.11 |  |
| IL-6 | 4.1 (0.0–20.1) | | 0.0 (0.0–8.7) | | | 0.17 |  |
| IL-7 | 5.4 (0.0–11.3) | | 1.8 (0.0–6.0) | | | 0.16 |  |
| IL-8 | 18.0 (11.0–38.6) | | 11.1 (5.0–17.6) | | | 0.003 |  |
| IL-12(p70) | 2.6 (0.0–13.7) | | 4.6(0.0–27.0) | | | 0.30 |  |
| IL-15 | 1.1(0.0–5.0) | | 0.8 (0.0–3.0) | | | 0.28 |  |
| IL-17 | 6.0 (1.4–20.0) | | 3.3(0.4–18.5) | | | 0.61 |  |
| EGF | 156.0 (70.2–298.4) | | 127.9 (68.3–216.7) | | | 0.28 |  |
| Eotaxin | 151.7 (96.3–176.0) | | 122.1 (89.4–150.4) | | | 0.18 |  |
| Fractalkine | 52.0 (12.8–95.9) | | 32.5 (0.0–69.4) | | | 0.40 |  |
| G-CSF | 11.1 (2.6–31.3) | | 13.0 (0.0–25.0) | | | 0.45 |  |
| GM-CSF | 8.7 (4.4–16.7) | | 8.7 (2.9–22.6) | | | 0.89 |  |
| IFN-γ | 19.8 (3.8–38.9) | | 11.7 (1.1–33.2) | | | 0.26 |  |
| IP-10 | 401.8 (266.4–522.5) | | 322.3(216.3–515.8) | | | 0.28 |  |
| MCP-1 | 510.0 (331.1–725.0) | | 469.8 (321.0–573.0) | | | 0.45 |  |
| MIP-1α | 9.7 (0.0–20.1) | | 0.0 (0.0–13.1) | | | 0.14 |  |
| MIP-1β | 45.9 (27.4–71.8) | | 49.2 (32.7–64.0) | | | 0.97 |  |
| TGF-α | 2.6 (1.3–4.8) | | 1.8 (0.1–3.7) | | | 0.25 |  |
| TNF-α | 16.2 (10.3–24.1) | | 16.2(10.5–23.1) | | | 0.49 |  |
| FGF-2 | 66.9 (42.5–88.6) | | 55.4 (32.1–120.3) | | | 0.64 |  |
| GRO | 1275.7 (865.8–1484.6) | | 908.8 (567.9–1295.3) | | | 0.01 |  |
| IFN-α2 | 30.4 (7.1–49.8) | | 7.1 (0.0–72.6) | | | 0.38 |  |
| IL-1ra | 11.9 (0.0–167.9) | | 0.0(0.0–23.8) | | | 0.03 |  |
| MDC(CCL22) | 697.4 (526.0–886.2) | | 761.7 (565.1–1021.5) | | | 0.42 |  |
| sCD40L | 5057.3 (3171.4–6535.8) | | 4640.6 (2839.5–5651.9) | | | 0.25 |  |
| VEGF | 123.1 (84.9–219.6) | | 96.4(38.5–259.3) | | | 0.75 |  |

Values are the median (interquartile range) pg/ml. Twenty-nine of 38 cytokine profiles were measurable in this study. EGF: epidermal growth factor, FGF-2: fibroblast growth factor, G-CSF: granulocyte-colony stimulating factor, GM-CSF: granulocyte macrophage-colony stimulating factor, GRO: growth-related cytokine , IFN: interferon, IL: interleukin, IP: induced protein, MCP: monocyte chemotactic protein, MDC: myeloid dendritic cells, MIP: macrophage inflammatory protein, TGF: transforming growth factor, TNF: tumor necrosis factor-alpha, VEGF: vascular endothelial growth factor.
