# Supplementary material for: The prevalence of insomnia and restless legs syndrome among Japanese outpatients with rheumatic disease: A cross-sectional study
Source: PLoS One. 2020 Mar 20;15(3):e0230273. doi: 10.1371/journal.pone.0230273 (PMC7083624; doi:10.1371/journal.pone.0230273)
Supplement: Attachment — Submitted filename: Response to reviewers_FINAL VERSION.docx [file pone.0230273.s006.docx]

**Re: PONE-D-19-29396, entitled: "The prevalence of insomnia and restless legs syndrome among Japanese outpatients with rheumatic disease: A cross-sectional study" by Urashima et al.**

**RESPONSE TO THE ACADEMIC EDITOR'S COMMENTS**

We thank the academic editor for the insightful comments on our manuscript. The comments have helped us significantly improve the manuscript. Based on these comments and suggestions, we have made careful modifications to the original manuscript. The changes are shown in the marked copy (highlighted in yellow). We hope that the revised manuscript can be published in the PLOS One following these significant changes.

1. Paragraph on patients, page 5-6.Please, specify the "initial study criteria", and in particular the eligibility criteria and the sources and methods of selection of participants, according to the STROBE checklist. Were the patients consecutive?

**Response 1:** We collected the consecutive data of 121 patients with rheumatic disease treated between August 2017 and May 2018 at the outpatient clinic at Nagasaki University Hospital. The quality of the studies was assessed on the basis of elements from the STROBE checklist for cross-sectional studies. We added the following text, starting on page 6:

"The eligibility criteria for patients participating in this study were: (1) having a clinical diagnosis of RA, systemic lupus erythematosus (SLE), mixed connective tissue disease (MCTD), systemic sclerosis, vasculitis, osteoarthritis, dermatomyositis, or spondyloarthritis by a rheumatologist; (2) a current user of at least prednisolone or an immunosuppressant according to their medical records; (3) >20 years old, and 4) able to provide informed consent. The exclusion criteria were: (1) the presence of any comorbidity of a medical or psychological/psychiatric condition or treatment revealed by a review of the patient's previous or outside medical records, in the opinion of the Principal Investigator, and (2) being unwilling to participate in a research study or provide research samples or data."

2. please, present differences with P >= 0.05 as non-significant, rather than as trends or tendencies. Please, provide a reference for the Classification and Regression Trees (CART) algorithm. Please, avoid making unsupported inferences about the significance of differences between your study sample and the general population (in this respect, cf. also the comment by reviewer 3 on the lack of a control group in your study).

**Response 2:** Thank you for your suggestion. We modified the expression from being a tendency to no significant difference. We added the reference for the CART algorithm (Breiman et al. 1984) in the Statistical Analysis section.

We agreed with your comment regarding including a randomly selected control sample. Unfortunately, we were unable to present the control data in this study because we did not envisage the inclusion of healthy individuals in our original research plan. We now note this as a study limitation. We avoided making unfounded inferences about the importance of differences between the study sample and the general population.

3. In the Discussion section, please describe your efforts to address potential sources of bias, as per the STROBE checklist, addressing in particular confounding variables, lack of generalizability, selective reporting, post hoc analyses, and data dredging. In the paragraph on limitations (page 18), please clarify or correct the sentence "We examined a small number of outpatients (n=41) treated at a single center".

**Response 3:** In accord with this comment, we added the following text to the Discussion section.

"Second, there was a potential source of bias at the baseline including confounding (per the STROBE checklist). Because the number of RLS patients in this study was small and a multivariate analysis could not be performed, the adjustment for confounding factors was insufficient. There was also a lack of generalizability, because all of the rheumatic diseases were included for the analyses, and it is difficult to generalize in regard to disease backgrounds and treatments associated with insomnia and RLS. Additional similar investigations based on each rheumatic disease would be informative.

Third, patients with high disease activity could not be included because this study was of outpatients with stable symptoms, and thus patients with relatively mild symptoms of insomnia and RLS might have participated. However, all of the data are presented in order to avoid selective reporting. In this study, an analysis was performed based on a hypothesis, and there is no evidence of data dredging. Further prospective studies of larger multicenter populations are required to clarify and sufficiently adjust the analyses for RLS and related factors in rheumatoid diseases, including confounding factors."

We also modified the number of outpatients to "n=70".

4. Please, cite and discuss the following reference: Mod Rheumatol. 2013 Jul;23(4):705-8. doi: 10.1007/s10165-012-0714-x. Epub 2012 Jul 14, which is related to RLS and rheumatic diseases in Asia.

**Response 4:** We now cite the reference. In addition, this is the first study evaluating the prevalence of RLS among patients with rheumatic diseases in Asia. Therefore, we revised the description in the Introduction and Discussion sections.

5. The last paragraph of the Discussion (page 19) needs be rewritten. The sentence "The patients ... were associated with RLS" is written incorrectly. The following sentence would have been valid even irrespective of the present manuscript, and would thus seem to support the third reviewer's concern that this manuscript is not characterized by a sufficient level of novelty for the advancement of our knowledge in this field. Please, elaborate and clarify.

**Response 5:** Thank you for your valuable comments. We modified the last paragraph of the Discussion.

6. Please, increase the resolution of supplementary figure 2.

**Response 6:** We increased the resolution of Supplementary Figure S2.

7. PLOS journals require authors to make all data necessary to replicate their study's findings publicly available without restriction at the time of publication (cf. https://journals.plos.org/plosone/s/data-availability). You are therefore required to submit the values behind the median and interquartile ranges and other measures reported and the values used to build graphs, either in a public repository (recommended) or as supplementary files.

**Response 7:** All data generated or analyzed during this study are included in this manuscript and its supplementary files.

**Re: PONE-D-19-29396, entitled: "The prevalence of insomnia and restless legs syndrome among Japanese outpatients with rheumatic disease: A cross-sectional study" by Urashima et al.**

**RESPONSE TO COMMENTS FROM** **REVIEWER 1**

We thank the Reviewer for the valuable suggestions that have helped us improve our manuscript considerably. As indicated in the responses below, we have taken all of the Reviewer's input into account in the revised version of our manuscript. Text added to the manuscript is highlighted in yellow. Our specific responses to the comments are listed below.

1. Inclusion and exclusion criteria should be detailed. STROBE guidelines must be explained or related to a citation.

**Response 1:** We have rewritten the inclusion and exclusion criteria for patients participating in this study in the Materials and Methods section. We cited the journal related to the STROBE guidelines as ref. 18.

2. The authors correctly approached the queries by an adequate analysis of both scores and laboratory data although the sample size is quite small to generalize the results. The authors did a good examination of the literature and clearly displayed limits of the study.

**Response 2:** The estimated 95% confidence interval for the prevalence of RLS among our participants was from 6.1% to 22.5%. The estimation is wide due to the small sample size. We have added this information to the study limitations text.

3. The study is well written and contextualized, but not particularly original in the hypothesis and therefore in the conclusions. In fact, these are clinic-lab data observations that confirm the general literature data applied to rheumatoid arthritis

**Response 3:** We thank the reviewer for this comment. Our study's primary endpoint was the prevalence of RLS in rheumatic diseases such as rheumatoid arthritis. Risk factors for RLS in patients with rheumatic disease were examined as a secondary endpoint of the study. There are no reports of similar studies in Japan, and we believe there is novelty in this respect.

**Re: PONE-D-19-29396, entitled: "The prevalence of insomnia and restless legs syndrome among Japanese outpatients with rheumatic disease: A cross-sectional study" by Urashima et al.**

**RESPONSE TO COMMENTS FROM** **REVIEWER 2**

<*Major comments*>

1. The definition of insomnia (primary endpoint of this study) is not clear. Did patients had face to face interview? Was ICSD-3 or DSM-5 criteria used for the diagnostic of insomnia?

**Response 1:** We did not diagnose chronic insomnia disorders based on ICSD-3 or DMS-5. We only evaluated the prevalence of insomnia symptoms and the severity of insomnia symptoms among patients with rheumatic diseases. Therefore, we revised the related descriptions in the Methods, Results, and Discussion sections.

2. In the method section, authors defines clinically severe insomnia with threshold of insomnia severity index (ISI) above 10/28, instead of >14/28, the validated cut-off for moderate insomnia, and the cutoff of 22/28 for severe insomnia. Can authors justify why they used different ISI cutoff for this study?

**Response 2:** The pathological cutoff value for insomnia based on the Japanese version of ISI, which has been validated by Munezawa et al., had been reported as above 10/28. We now cite the reference and revised the description in the Methods section.

3. The international RLS study group rating scale (IRLSQ) is worldwide used to assess RLS severity. Did authors assess severity with IRLSQ? If not available, is there any data on RLS severity assessment (Clinical global impression)?

**Response 3:** We did not assess RLS severity using the IRLSQ. Instead, the items about the frequency of symptom occurrence and distressing symptoms in the CH-RLSq13 were used to assess clinically significant RLS (referred to as "RLS sufferers"): the frequency item was ≥ 2-3 days/week, and the distress item was either moderate or extreme distress. Only one RLS patient met the criteria for "RLS sufferers" as described in the Discussion section.

<*Minor comments*>

4. In the introduction, it's stated that sleep deprivation can cause the onset of autoimmune diseases, however, the study cited is on animal model, can authors provide other clinical studies?

**Response 4:** We changed this to other clinical studies (Hsiao YH 2015, Chandrasekhara PK, 2009).

5. The presence of RLS was well done, as RLS was screened with questionnaire, and the diagnostic confirmed by physician, in this population with chronic pain complain, a condition that can mimic RLS symptoms. Can authors provide how many patients met the five essential criteria, and how many have RLS Diagnostic?

**Response 5:** We interviewed all participants who answered "yes" to our survey question about the main symptoms of RLS: " Do you have, or have you had, recurrent uncomfortable feelings or sensations in your legs while you are sitting or lying down?" and/or "Do you, or have you had, a recurrent need or urge to move your legs while you were sitting or lying down?" Ten participants met the five essential criteria, and the RLS-related symptoms among all of them were not explained by rheumatic disease-related arthralgia or other conditions. Therefore, 10 participants were diagnosed as having RLS.

6. What was the age at RLS onset and age at daily RLS symptoms? Some patients didn't had daily RLS symptoms, can authors provide the frequency of RLS for each patients? Did authors considered patients with less than 3 time a week of RLS symptom as sufferer?

**Response 6:** The median age (minimum – maximum) at RLS onset was 40 (15–60) years. Five of 10 patients with RLS developed RLS after the diagnosis of rheumatic disease; in three of them the RLS and rheumatic disease had developed at almost the same time; in one patient the RLS had developed before the diagnosis of rheumatic disease, and the onset of RLS was not known in the fifth patient. Nine patients had experienced RLS symptoms <2 days/week in the prior 12 months, and only one patient experienced RLS symptoms 4–5 days per week. Therefore, one patient was considered an RLS sufferer. We have added these findings to the Results section.

7. In case of ferritin below 75µg/l, RLS is considered as secondary to iron depletion. Can authors provide how many patients with RLS were below this cutoff ? is there any difference between RLS and no-RLS patients ?

**Response 7:** In the present study, 44 (64.7%) of 68 patients whose ferritin was measured were found to be <75 ng/ml. In the RLS and non-RLS groups, ferritin < 75 mg/ml was present in 8 of 10 patients (80.0%) and in 36 of 58 patients (62.1%), respectively, showing no significant difference between the two groups (p=0.48).

8. Patients with comorbid RLS and RD were younger than those without RLS. However, RLS frequency increase with age in the general population. How authors explain such result?

**Response 8:** We strongly appreciate the reviewer's comment on this point. We added the following text to the Discussion section:

" The age of the present RLS group was significantly lower than that of the non-RLS group (p=0.02). In this study, five of the 10 patients in the RLS group developed RLS before the age of 45 years. It was reported that early-onset RLS (occurring before the age of 45 years) has a high incidence of family history [39, 40]. In the present examination, one or more genetic factors may be involved with the earlier onset of RLS; however, our patients' family histories of RLS were not investigated sufficiently."

9. In the discussion section, there is mistake : "Interestingly, five of the 10 patients in the present RLS group had developed RLS after the diagnosis of rheumatic disease: one patient had developed RLS during almost the same period, and three patients developed rheumatic disease before the onset of RLS (the time course of the fifth RLS patient was unknown)."

How many patients have RLS before RD diagnostic, and how many after?

**Response 9:** We modified the sentence as follows on page 22:

"Interestingly, half of our patients with RLS developed RLS after the diagnosis of rheumatic disease; in three of these five patients, the RLS and rheumatic disease had developed almost simultaneously; in one patient, the RLS developed before the diagnosis of rheumatic disease, and in the remaining patient, the onset of RLS was unknown."

10. In the discussion section, this sentence is not clear: "The correlation of pairs of cytokines/chemokines/growth factors showed different patterns between the PSL ≥3 mg/day group and the PSL <3 mg/day group. The correlation of pro- and anti-inflammatory cytokines in serum varied between the PSL ≥3 mg/day and <3 mg/day groups, and these variations may have affected the disease activity and mechanism of RLS."
Please, can authors explain with more details what is the clinical implication of such finding?

**Response 10:** We revised the sentence as follows on page 23:

" The correlations of pairs of cytokines/chemokines/growth factors showed different patterns between the PSL ≥3 mg/day group and the PSL <3 mg/day group. The cytokines/chemokines/growth factors including pro-inflammatory cytokines (IL-2, IL-6, IL-15, IFN-γ) were generally lower and correlated with an anti-inflammatory cytokine (IL-1ra) with PSL <3 mg/day group compared to the PSL ≥3 mg/day group. This result may indicate that an anti-inflammatory cytokine might exert control in the PSL <3 mg/day group as a host defense mechanism. Accordingly, our PSL ≥3 mg/day and <3 mg/day groups differed with elevated downstream levels of pro-inflammatory cytokines/chemokines/growth factors, and these variations may have affected the disease activity and mechanism of RLS."

**Re: PONE-D-19-29396, entitled: "The prevalence of insomnia and restless legs syndrome among Japanese outpatients with rheumatic disease: A cross-sectional study" by Urashima et al.**

**RESPONSE TO COMMENTS FROM** **REVIEWER 3**

We thank the Reviewer for the insightful comments on our manuscript. The comments have helped us significantly improve the manuscript. Based on these comments and suggestions, we have made careful modifications to the original manuscript. The changes are shown in the marked copy (highlighted in yellow). We hope that the revised manuscript can be published in the PLOS One following these significant changes.

1. The sample size is quite small for a study of this nature:

**Response 1:** Indeed, the sample size of the present study is relatively small considering the extent of the study area. We added the following text to the limitations section on page 25:

"In addition, the total sample size at the start of the study was not determined, because the significance of what our findings revealed related to our outcome measurement (efficacy of the protocol or proposed technique) and the comparisons thereof is exploratory and not confirmative."

2. The authors should have included a control group randomly selected from the general population of the same geographical region;

**Response 2:** We agree regarding the randomly selected control sample, but unfortunately, we were unable to present the control data in this study because we did not envisage the inclusion of healthy individuals in our original research plan. We now note this as a limitation of the study.

3. The English form needs improvement.

**Response 3:** We have had the manuscript rewritten by an experienced scientific editor who improved the grammar and stylistic expression of the text.

**Re: PONE-D-19-29396, entitled: "The prevalence of insomnia and restless legs syndrome among Japanese outpatients with rheumatic disease: A cross-sectional study" by Urashima et al.**

**RESPONSE TO COMMENTS FROM** **REVIEWER 4**

*Major issue*:

The paper is written in a form of English that even though are grammatically correct, do not match a scientific way of writing (e.g. we also analyzed, we next compared, etc)

The discussion is very descriptive with no information regarding potential mechanisms and physiological explanations.

Authors need to critically review their data suggesting potential mechanisms of causes

**Response:** We have had the manuscript rewritten by an experienced scientific editor who improved the grammar and stylistic expression of the paper. In this study, we clarified that two risk factors for RLS in the patients with rheumatic disease were the internal dose of PSL and a lower level of TSAT. Previous reports suggested that a lower level of TSAT was associated with RLS, because impaired iron utilization by inflammation induced a lower level of TSAT. We also showed that the pattern of cytokines expressed and the relationship between cytokines differed between the patient groups with PSL ≥ 3 mg/day and PSL < 3 mg/day, demonstrating the mechanisms by which inflammation and autoimmune phenomena contribute to the development of RLS. We revised the Discussion section based on this information.
