# Supplementary material for: The prevalence of insomnia and restless legs syndrome among Japanese outpatients with rheumatic disease: A cross-sectional study
Source: PLoS One. 2020 Mar 20;15(3):e0230273. doi: 10.1371/journal.pone.0230273 (PMC7083624; doi:10.1371/journal.pone.0230273)
Supplement: Attachment — Submitted filename: Response to reviewers_R1_022120_FINAL VERSION.docx [file pone.0230273.s007.docx]

**Re: PONE-D-19-29396R1, entitled: "The prevalence of insomnia and restless legs syndrome among Japanese outpatients with rheumatic disease: A cross-sectional study" by Urashima et al.**

**RESPONSE TO THE ACADEMIC EDITOR'S COMMENTS**

We sincerely thank the Academic Editor for the insightful comments on our manuscript. The comments have helped us significantly improve the manuscript. Based on these comments and suggestions, we have made careful modifications to the original manuscript. The changes are shown in the marked copy (highlighted in yellow). We hope that the revised manuscript can be published in the PLOS ONE following these significant changes.

- 1. Consecutive enrollment should be mentioned in the manuscript text and not only in the rebuttal.

**Response 1:** We apologize for not including this in the text of the previous revision. We added the following text, starting on line 70:

"We collected the consecutive data of 121 patients with rheumatic disease treated between August 2017 and May 2018 at the outpatient clinic at Nagasaki University Hospital. The quality of the studies was assessed on the basis of elements from the STROBE checklist for cross-sectional studies [19]."

- 2. The meaning of the sentence "There was also a lack of generalizability, because all of the rheumatic diseases were included for the analyses, and it is difficult to generalize in regard to disease backgrounds and treatments associated with insomnia and RLS" is unclear. Generalizability from the STROBE checklist perspective refers to external validity. How does this relate to "generalize in regard to disease backgrounds and treatments associated with insomnia and RLS"?

**Response 2:** We rewrote the relevant text as follows, starting at line 358:

"In addition, patients with various rheumatic diseases such as RA and SLE were included in this study, and thus the patients' background characteristics such as being at a susceptible age for a disease and the amount of PSL and concomitant immunosuppressants varied depending on the rheumatic disease. The study population was thus not homogeneous with respect to the patients' backgrounds and treatments, which are likely to affect insomnia and RLS."

- 3. The meaning of the sentence "In addition, the total sample size at the start of the study was not determined, because the significance of what our findings revealed related to our outcome measurement (efficacy of the protocol or proposed technique) and the comparisons thereof is exploratory and not confirmative" is even less clear. Please, reword, and make specific reference to statistical power analysis.

**Response 3:** We rewrote the relevant text as follows, at line 350:

"Assuming a prevalence of 0.25 of RLS among patients with rheumatic diseases, a sample size of 199 patients was regarded as sufficient to measure this proportion with a desired width of a 95% confidence level of ±0.05. This was an exploratory study, and we did not determine the number of samples required at the start of the study because the prevalence of RLS in Japanese rheumatic diseases is unknown."

- 4. In the conclusions, please make it explicit that the results mentioned from line 388 onwards are to be considered as exploratory due to the limitations of the study.

**Response 4:** We rewrote the relevant text as follows, at line 375:

"The prevalence of RLS in this series of patients with rheumatic diseases was 14.3%, and we consider this value as simply exploratory due to the limitations of the study."
