# Supplementary material for: The prevalence of insomnia and restless legs syndrome among Japanese outpatients with rheumatic disease: A cross-sectional study
Source: PLoS One. 2020 Mar 20;15(3):e0230273. doi: 10.1371/journal.pone.0230273 (PMC7083624; doi:10.1371/journal.pone.0230273)
Supplement: Attachment — Submitted filename: Response to reviewers_R2_022520_FINAL VERSION.docx [file pone.0230273.s008.docx]

**Re: PONE-D-19-29396R2, entitled: "The prevalence of insomnia and restless legs syndrome among Japanese outpatients with rheumatic disease: A cross-sectional study" by Urashima et al.**

**RESPONSE TO THE ACADEMIC EDITOR'S COMMENTS**

We sincerely thank the Academic Editor for the insightful comments on our manuscript. The comments have helped us significantly improve the manuscript. Based on these comments and suggestions, we have made careful modifications to the original manuscript. The changes are shown in the marked copy (highlighted in yellow). We hope that the revised manuscript can be published in the PLOS ONE following these significant changes.

-1. In particular, the text at line 69 ("We collected the consecutive data of 121 patients...") is unclear. Does the adjective "consecutive" refer to the data or to the patients? This has implications (cf, eg, paper PMID 23846607). Please, reword and clarify.

**Response 1:** We rewrote the relevant text as follows, starting at line 69:

“We collected 121 consecutive patients with rheumatic disease treated between August 2017 and May 2018 at the outpatient clinic at Nagasaki University Hospital.”

-2. Moreover, the revised text at lines 189-191 and 342-347 appears statistically inconsequent (cf, eg, papers PMID 11560206 and https://doi.org/10.1080/19312450701641375). I suggest that you delete this text and simply modify the text at lines 341-342 as follows: "The limitations of our study deserve some discussion. First, the study population consisted of a small number of outpatients (n=70) treated at a single center, with no a-priori statistical power analysis."

**Response 2:** According to the comment, we deleted the relevant text at lines 189-191 and 342-347 and modified the text starting at line 339 as follows:

"The limitations of our study deserve some discussion. First, the study population consisted of a small number of outpatients (n=70) treated at a single center, with no a-priori statistical power analysis."
